# SARS-CoV-2 Infection Induces Psoriatic Arthritis Flares and Enthesis Resident Plasmacytoid Dendritic Cell Type-1 Interferon Inhibition by JAK Antagonism Offer Novel Spondyloarthritis Pathogenesis Insights

**DOI:** 10.3389/fimmu.2021.635018

**Published:** 2021-04-15

**Authors:** Qiao Zhou, Jayakumar Vadakekolathu, Abdulla Watad, Kassem Sharif, Tobias Russell, Hannah Rowe, Almas Khan, Peter A. Millner, Peter Loughenbury, Abhay Rao, Robert Dunsmuir, Jake Timothy, Giovanni Damiani, Paolo D. M. Pigatto, Piergiorgio Malagoli, Giuseppe Banfi, Yasser M. El-Sherbiny, Charlie Bridgewood, Dennis McGonagle

**Affiliations:** ^1^ Department of Rheumatology and Immunology, Sichuan Provincial People’s Hospital, University of Electronic Science and Technology of China, Chengdu, China; ^2^ Chinese Academy of Sciences Sichuan Translational Medicine Research Hospital, Chengdu, China; ^3^ Leeds Institute of Rheumatic and Musculoskeletal Medicine (LIRMM), University of Leeds, Leeds, United Kingdom; ^4^ Department of Biosciences, School of Science and Technology, Nottingham Trent University, Nottingham, United Kingdom; ^5^ Leeds Teaching Hospitals NHS Trust, Leeds, United Kingdom; ^6^ Department of Neurosurgery, Leeds Centre for Neurosciences, Leeds General Infirmary, Leeds, United Kingdom; ^7^ Clinical Dermatology, IRCCS Istituto Ortopedico Galeazzi, Milan, Italy; ^8^ Department of Biomedical, Surgical and Dental Sciences, University of Milan, Milan, Italy; ^9^ Dermatology Unit, Azienda Ospedaliera San Donato Milanese, Milan, Italy; ^10^ School of Medicine, Universitá Vita-Salute San Raffaele, Milan, Italy; ^11^ National Institute for Health Research (NIHR) Leeds Biomedical Research Centre (BRC), Leeds Teaching Hospitals, Leeds, United Kingdom

**Keywords:** plasmacytoid dendritic cells, interferon alpha, psoriatic arthritis, COVID-19, enthesis

## Abstract

**Objective:**

Bacterial and viral infectious triggers are linked to spondyloarthritis (SpA) including psoriatic arthritis (PsA) development, likely *via* dendritic cell activation. We investigated spinal entheseal plasmacytoid dendritic cells (pDCs) toll-like receptor (TLR)-7 and 9 activation and therapeutic modulation, including JAK inhibition. We also investigated if COVID-19 infection, a potent TLR-7 stimulator triggered PsA flares.

**Methods:**

Normal entheseal pDCs were characterized and stimulated with imiquimod and CpG oligodeoxynucleotides (ODN) to evaluate TNF and IFNα production. NanoString gene expression assay of total pDCs RNA was performed pre- and post- ODN stimulation. Pharmacological inhibition of induced IFNα protein was performed with Tofacitinib and PDE4 inhibition. The impact of SARS-CoV2 viral infection on PsA flares was evaluated.

**Results:**

CD45+HLA-DR+CD123+CD303+CD11c- entheseal pDCs were more numerous than blood pDCs (1.9 ± 0.8% vs 0.2 ± 0.07% of CD45+ cells, p=0.008) and showed inducible IFNα and TNF protein following ODN/imiquimod stimulation and were the sole entheseal IFNα producers. NanoString data identified 11 significantly upregulated differentially expressed genes (DEGs) including TNF in stimulated pDCs. Canonical pathway analysis revealed activation of dendritic cell maturation, NF-κB signaling, toll-like receptor signaling and JAK/STAT signaling pathways following ODN stimulation. Both tofacitinib and PDE4i strongly attenuated ODN induced IFNα. DAPSA scores elevations occurred in 18 PsA cases with SARS-CoV2 infection (9.7 ± 4 pre-infection and 35.3 ± 7.5 during infection).

**Conclusion:**

Entheseal pDCs link microbes to TNF/IFNα production. SARS-CoV-2 infection is associated with PsA Flares and JAK inhibition suppressed activated entheseal plasmacytoid dendritic Type-1 interferon responses as pointers towards a novel mechanism of PsA and SpA-related arthropathy.

## Introduction

Entheses are locations where tendons or ligaments attach to the bone and inflammation of the enthesis (enthesitis) is an important and frequent manifestation of inflammatory musculoskeletal disease, especially the Spondyloarthritis (SpA) group of diseases, which includes ankylosing spondylitis (AS) and psoriatic arthritis (PsA) (1–3). Plasmacytoid dendritic cells (pDCs), are a rare immune cell subset that was first functionally characterized in the late 1990s (4) that upon toll-like receptor (TLR)-7 and TLR-9 stimulation, which recognize RNA and DNA respectively, are known to secrete type-I interferons (IFN) and other inflammatory cytokines such as TNF (5, 6). Moreover, pDCs have been implicated in psoriasis immunopathology whereby cutaneous pDCs orchestrate disease development through IFNα induction culminating in psoriasis development (4, 7, 8). Bacterial or viral infection or vaccinations have been linked to PsA and SpA like disease (7, 9, 10).

The IL-17 axis and TNF cytokine pathways are central to the pathogenesis of PsA and SpA, but the emerging JAK inhibition in PsA and AS do not directly impact on the TNF/IL-17 pathways (11). However, the JAK-STAT pathway is a critical regulator of pDC cytokine production, including TNF and interferon. Nevertheless, direct evidence for this pathway as a potential human SpA initiator is lacking. During the global COVID-19 virus pandemic, arthralgia has been reported, raising the possibility of a poorly defined entheseal mechanism (12, 13).

We previously found evidence for a population of entheseal resident pDCs by phenotypic criteria (14). Herein, we investigated immune responses of entheseal pDCs following TLR7 and TLR9 stimulation and the potential modulation of this response by emergent SpA therapeutics and also whether the SARS-CoV-2 RNA virus, with potent TLR7 agonist properties was associated with PsA disease flares.

## Materials and Methods

### Enthesis Samples

Normal interspinous process was obtained from 54 patients (20 men and 34 women; age 53.6 ± 23.2 years) who were undergoing spinal decompression or corrective scoliosis surgery. Peripheral blood was also collected from these patients and healthy volunteers (n=5). The enthesis was subsequently separated into peri-entheseal bone (PEB) and the entheseal soft tissue (EST). PEB was chosen for the following experiments because cells were more numerous in PEB than in ST. Mononuclear cells were isolated from PEB by mechanical digestion, as previously indicated (14, 15). The study protocol of the current investigation was approved by North West-Greater Manchester West Research Ethics Committee.

### Phenotyping of Entheseal pDC and Intracellular Measurement of TNF Following ODN or Imiquimod Stimulation

Following digestion, 5×10^6^ PEB cells were incubated in RPMI (Sigma-Aldrich) containing 10% FBS (Gibco) and 1% p/s (Penicillin-Streptomycin). Cells were stimulated with CpG oligodeoxynucleotides (ODN 2216, InvivoGen, 20 μg/mL) or imiquimod (30 μM, Cayman Chemical) for 2 hr. Cells were subsequently incubated for 12 hr in the presence of Golgi Plug (BD Biosciences). Following this, cells were blocked in 10% mouse serum and 1% human IgG buffer prior to incubation with antibodies. Cells were then stained extra and intracellularly using the Intraprep staining kit (Beckman Coulter) according to the manufacturer’s protocol. Cells were stained using anti-CD45, CD3, CD14, CD19, CD56, CD11c, HLA-DR, CD123, CD303 and TNF (additional details on the antibodies and processes used available upon request from the corresponding author). Cells were analyzed using LSRII (BD Biosciences) and FlowJo software (Tree Star Software).

### Isolation of pDC and Tomographic Microscopy

Following digestion, pDCs were selected using CD304 (BDCA-4)-Biotin (Miotenyl Biotec, 2 μl per 10×10^6 cells) using LS columns (Miotenyl Biotec) according to the manufacturer’s protocol. Cells were subsequently separated into pDCs and non-pDCs. Isolated entheseal pDCs were seeded into 35-mm tissue culture dish at 15,000 cells/dish. The dish was then placed on a holotomographic microscope (3D Explorer, NanoLive, Lausanne, Switzerland) equipped with a 60X objective, and images were taken at ×600 magnification.

### Stimulation of pDCs and Quantification of IFNα by ODN

Due to sample size variance and resultant cell yields, different pDC numbers were isolated and seeded per experiment (between 12000 and 50000 per well) with IFNα secretion being expressed as pg/cell. During method optimization we noted that ODN was a much more potent inducer of entheseal pDC IFNα production than imiquimod (**Supplementary Figure1B**). Consequently, further *in vitro* studies were carried out on ODN stimulated cells whereby cells were stimulated with 20 μg/ml ODN for 24 hrs. Following this, supernatant was harvested. IFNα (all isoforms) were measured using ELISA (PBL Assay Science). The cells were harvested and RNA was isolated using PicoPure RNA isolation kit (ThermoFisher) and complementary DNA was synthesized using a high-capacity reverse transcription kit (ThermoFisher). Quantitative real-time polymerase chain reaction (PCR) with an ABI 7500 thermocycler (Applied Biosystems) was performed to measure IFNα gene expression using Taqman gene expression assay and universal Taqman master mix (both Thermofisher). Expression levels of target gene were calculated relative to expression of the housekeeping gene HPRT1.

### Antagonism of IFNα Secretion

After digestion, 1×10^5^ cells/ml entheseal cells were seeded into 96-well plate. Cells were stimulated with ODN as before, with and without tofacitinib 1 μM (Pfizer), PDE4i/Rolipram 100µM (Cayman Chemical) or Methotrexate 5 mg/ml (Cayman Chemical) for 24 hr. DMSO 0.1% served as a solvent control. IFNα was quantified by ELISA as before.

### Nanostring Analysis of Entheseal and Blood pDCs Upon ODN Stimulation

Post ODN stimulation, RNA was extracted from enriched entheseal pDCs and matched blood cells as described above (each n=5). Immune related gene expression was assessed using a predesigned human autoimmune profiling panel established by Nanostring consisting of 770 immune related (genes involved in autoimmune, chronic inflammatory, and aberrant immune response diseases) and 20 housekeeping genes. All RNA samples were quality controlled using Nanodrop 8000. 100ng of total RNA from each sample was used for setting up the NanoString probe PCR hybridization (24 hrs) at 65°C. Following hybridization, excess probes were removed using nCounter Prep Station and magnetic beads, hybridized mRNA/probe were immobilized on a streptavidin-coated cartridge. The processed cartridges were scanned using an nCounter digital analyzer platform (nCounter^®^ FLEX Analysis System) for generation of the raw data with a high-resolution scan (555 fov). Raw data were processed with nSolver Analysis Software (V.4.0), imaging quality control (QC), mRNA positive control QC and normalization QC checked, and all the samples were within the quality parameters of NanoString gene expression assays. Differential gene expression analysis was performed using nSolver advance analysis module V. 2.0.

### Bioinformatics Analysis

Nanostring data was analyzed using nSolver 4.0 and plot using Graphpad 8.0. Log_2_|fold change| >1 and Benjamini-Yekutieli p-value (FDR) <0.05 were considered statistically significant. Protein-protein interaction (PPI) network was analyzed using online STRING database (https://string-db.org/). Pathways mapping was generated by ingenuity pathway analysis (IPA, QIAGEN Inc.) (16). IPA was used to predicted possible upstream regulators of the proteins in this study, which were assigned as inhibited or activated according to Z-score, a statistical result of differential gene expression according to the fold changes (17). Enrichment is determined by significance overlap of genes known to be in this pathway according to the knowledge base and genes that are regulated in the dataset. Significance is measured by Fisher exact test by p-value.

### Assessment of Disease Flares in PsA Post COVID-19 Infection

SARS-CoV2 virus is a single-stranded RNA virus that stimulates TLR7 pathways. To provide further support for the potential relevance of viral infection in SpA related disease, 18 PsA patients (Classification Criteria for Psoriatic Arthritis (CASPAR) score ≥3) with a confirmed COVID-19 diagnosis by nasopharyngeal PCR were evaluated for PsA disease flare between February and April 2020. The disease activity in psoriatic arthritis (DAPSA) scores including swollen and tender joints count, were collected at three times points: T0-last most recent measurement before infection; T1-during infection, whilst the patient was stable; T2-post infection, following 2 negative nasopharyngeal specimen tests. Patient demographic is reported in **Table 1** (**Supplementary Material**).

**Table 1:** Basic demographic information for PsA patients.

Gender	Age (years)	BMI (kg/m^2^)	PsA duration in years	Current treatment	COVID treatment	Time between COVID infection and flare (days)
M	65	28	8	Ustekinumab	Oxygen, Lopinavir-ritonavir 400mg twice, Hydroxychloroquine 800mg/die, Prednisone 25 mg	13
M	53	29	11	Secukinumab	Amoxicillin 1000mg twice per day, Paracetamol 1000mg, Oxygen, Lopinavir-ritonavir 400mg twice	19
F	48	31	16	Secukinumab	Oxygen, Lopinavir-ritonavir 400mg twice, Hydroxychloroquine 800mg/die, Doxycycline 100 mg, Azithromycin 500 mg	21
M	67	27	14	Etanercept	Oxygen, Lopinavir-ritonavir 400mg twice, Hydroxychloroquine 800mg/die	17
F	61	28	12	Ustekinumab	Oxygen, Lopinavir-ritonavir 400mg twice, Hydroxychloroquine 800mg/die, Prednisone 25 mg	19
F	53	31	17	Adalimumab	Amoxicillin 1000mg twice per day, Paracetamol 1000mg, Oxygen, Hydroxychloroquine, Doxycycline 100	23
F	58	27	11	Ustekinumab	Doxycycline, Hydroxychloroquine, Lopinavir-ritonavir 400mg twice	14
M	45	26	13	Adalimumab	Oxygen, Lopinavir-ritonavir 400mg twice, Amoxicillin 1000mg, Prednisone 25 mg	12
M	57	27	12	Ustekinumab	Oxygen, Lopinavir-ritonavir 400mg twice, Hydroxychloroquine 800mg/die, Prednisone 25 mg	18
F	66	28	17	Adalimumab	Oxygen, Lopinavir-ritonavir 400mg twice, Hydroxychloroquine 800mg/die, Doxycycline	21
M	43	26	15	Etanercept	Hydroxychloroquine, Amoxicillin	15
F	54	28	16	Ustekinumab	Lopinavir-ritonavir 400mg twice, Hydroxychloroquine	19
F	69	27	9	Secukinumab	Oxygen, Lopinavir-ritonavir 400mg twice, Hydroxychloroquine 800mg/die, Amoxicillin 1000mg x2	16
M	45	28	6	Ixekinzumab	Oxygen, Lopinavir-ritonavir 400mg twice, Hydroxychloroquine 800mg/die, Prednisone 25 mg	17
F	37	28	13	Adalimumab	Oxygen, Lopinavir-ritonavir 400mg twice, Hydroxychloroquine 800mg/die, Prednisone 25 mg	14
F	71	29	7	Adalimumab	Hydroxychloroquine, Doxycycline	16
M	60	27	17	Ustekinumab	Hydroxychloroquine, Doxycycline	15
M	48	30	6	Adalimumab	Hydroxychloroquine, Doxycycline	17

M, male; F, female. BMI, body mass index.

### Statistical Analysis

Statistical significance was calculated using 2-tailed paired t-test unless stated. The analysis was performed using Prism software (GraphPad Software8.0). Error bars represent the standard error of the mean (SEM). p<0.05 was considered statistically significant.

## Results

### Human Entheseal pDCs Secrete TNF and IFNα After ODN Stimulation

**Figure 1:**
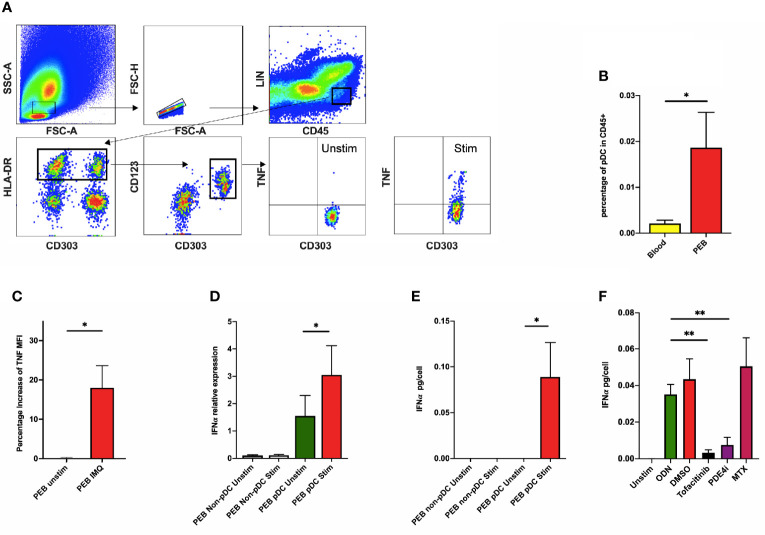
**(A)** Peri-entheseal bone (PEB) was separated from soft tissue and digested and pDC phenotype was confirmed using flow cytometry. Following stimulation with ODN, intracellular TNF was quantified using flow cytometry (last 2 graphs in **A**). **(B)** Flow cytometry showed that entheseal pDCs were more numerous than blood pDCs (1.86 ± 0.77% vs 0.21 ± 0.07% of CD45+ cells, n=5, p=0.008). **(C)** Mean percentage increase of TNF MFI also showed a significant increase after stimulation with imiquimod (18.03% vs 0.18%, n=4, p=0.047). Secretion of IFNα from pDCs after ODN stimulation was demonstrated by RT-PCR (n=8, **D**) and ELISA (n=6, **E**) respectively. The effect of different drugs on the expression of IFNα from entheseal cells was compared (n=4, **F**). IFNα from ODN stimulated: 0.035 ± 0.011 pg/cell, co-incubated with Tofacitinib: 0.003 ± 0.003 pg/cell, co-incubated with PED4i: 0.008 ± 0.008 pg/cell, co-incubated with MTX: 0.050 ± 0.031 pg/cell. LIN: lineage markers (CD3, CD56, CD19, CD14, CD11c), MFI, median fluorescence intensity; IMQ, imiquimod; Unstim, unstimulated; stim, stimulated. *p < 0.05, **p < 0.01.

Following entheseal digestion, pDCs were confirmed in PEB by flow cytometry by a CD45+HLA-DR+CD123+CD303+CD11c-Lin- phenotype (**Figure 1A**) and morphology is shown in **Supplementary Figure 1A**. Flow cytometry showed that pDCs were more numerous in PEB than in peripheral blood (1.86 ± 0.77% vs 0.21 ± 0.07% among CD45+ cells, n=5, p=0.008, **Figure 1B**). Entheseal cells stimulated with ODN showed upregulation of intracellular TNF (**Figure 1A**). TNF was also induced following imiquimod stimulation as determined by intracellular flow cytometry (n=4, **Figure 1C**).

Following isolation of both entheseal pDCs and non-pDCs and subsequent ODN stimulation, basal IFNα transcripts were detectable in unstimulated pDC (relative expression 1.552 ± 0.747, **Figure 1D**) but this was significantly higher following ODN stimulation (relative expression 3.053 ± 1.070, p=0.01, **Figure 1D**). At the protein level, IFNα was only detectable from the pDC stimulated fraction (0.089 ± 0.038 pg/cell, p=0.031, **Figure 1E**). Noting that total IFNα was secreted by entheseal pDCs, further experiments were undertaken on unsorted cells.

We investigated the effect of SpA disease-relevant compounds and their potential to modulate IFNα secretion. Both Tofacitinib (0.003 ± 0.002 pg/cell, p=0.008) and PDE4i (0.008 ± 0.004 pg/cell, p=0.002) significantly reduced ODN induced IFNα. Methotrexate however, had no effect on IFNα production (0.050 ± 0.016 pg/cell, p=0.24) (**Figure 1F**).

### DEG Pattern of Stimulated Entheseal pDCs and TNF Pathway

**Figure 2:**
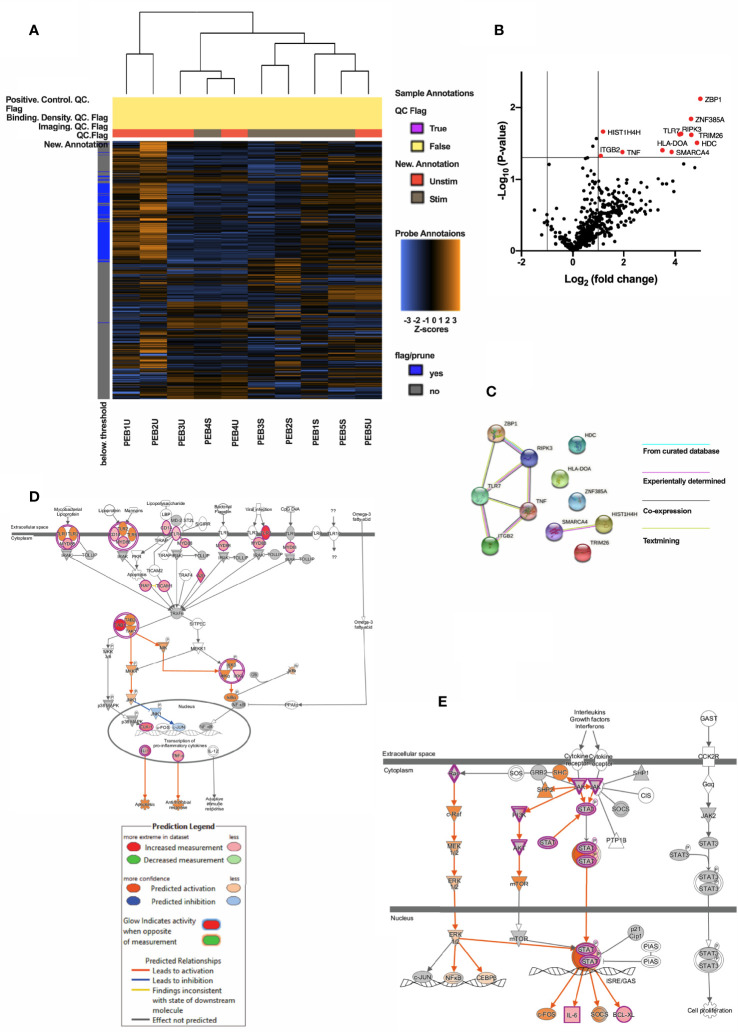
**(A)** Heatmap showing clustering of PEB stimulated pDCs and PEB unstimulated pDCs. **(B)** Volcano map showing 11 significantly upregulated genes (red, Log2(fold change) >1 and p<0.05) in stimulated vs unstimulated entheseal pDCs. Gene symbols were displayed if Log2|fold change| > 1. **(C)** PPI network of the 11 DEGs showing a main component consisted and 4 isolated nodes. Lines connecting two nodes represent protein-protein associations. Pathway mapping analysis of pDCs generated by IPA revealed the toll-like receptor signaling **(D)** and JAK/STAT signaling **(E)** among the topmost canonical pathways enriched upon treatment with ODN. Stimulation of TLRs leads to activation of MYD88 and finally transcription of pro-inflammatory cytokines including TNF **(D)**. Activation of JAK leads to activation of STAT or PI3K pathway, which is predicted by IPA **(E)**. Prediction legend is shared by 2D and 2E. Red & pink represents upregulation and intensity represents the relative magnitude of change in gene expression. Predicted activation indicated by orange color and blue for the predicted inhibition. Direct and indirect interactions are indicated by solid and dashed lines, respectively.

Following ODN stimulation of entheseal pDCs, using NanoString, hierarchically clustered heat map representation of global gene expression profiles showed that stimulated pDCs could be differentiated from the unstimulated samples, although heterogeneity might exist (**Figure 2A**). Eleven DEGs were significantly upregulated in the stimulated pDCs, including TNF (log_2_ fold change=1.95, p=0.042, **Figure 2B**). Protein-protein interaction (PPI) network was constructed from the 11 DEGs including 4 isolated nodes and 1 main connection component (**Figure 2C**). There were a total of 8 edges identified and PPI enrichment p-value was 0.004. The main component consisted of ZBP1, RIPK3, TNF, ITGB2 and TLR7. RIPK3 and TNF had the greatest degree of interaction (**Figure 2C**).

Canonical pathway analysis of the entheseal pDC genes upon ODN stimulation using IPA showed that pathways related to dendritic cell maturation (22 genes, z=4.416, p=1.59E-19, **Supplementary Figure 2**), NF-κB signaling (19 genes, z=3.3, p =5.5E-16, **Supplementary Figure 3**), toll-like receptor signaling (14 gens, z=2.714, p=1.65E-15, **Figure 2D**) and JAK/STAT signaling (8 genes, z= 2.828, p=3.05E-07, **Figure 2E**) were significantly activated.

### Increased DAPSA Following Sars-Cov-2 Infection

**Figure 3:**
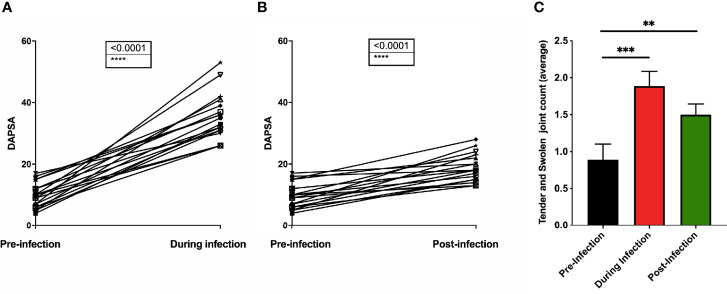
Psoriatic Arthritis patients had DAPSA score calculated both pre-COVID-19 infection and during infection **(A)** and post-infection **(B)**. The tender and swollen joint was scored, and the average calculated for pre-infection, during infection and post-infection **(C)**. n=18. **p < 0.01, ***p < 0.001 and *p* < 0.0001

18 PsA patients with confirmed COVID-19 showed increased DAPSA score following COVID-19 (**Figure 3A**, 9.7 ± 4.06 vs 35.3 ± 7.47 pre- and during infection, respectively, p<0.0001). Even post-infection, DAPSA scores remained higher (**Figure 3B**, 9.7 ± 4.06 *vs.* 18.8 ± 4.39 for pre- and post-infection, respectively, p<0.0001). With respect to swollen and tender joint count, statistically significant increases were shown for both during and post-infection (**Figure 3C**, 0.9 ± 0.9, 1.9 ± 0.8, 1.5 ± 0.6 for pre-, during, and post-infection, respectively. P=0.0005 for during- *vs.* pre-infection and p=0.002 for post- *vs.* pre-infection).

## Discussion

We report the presence of an enriched pDC population at the normal human enthesis. Upon relevant TLR pathway stimulation, we demonstrated inducible TNF and IFNα protein production and IPA analysis showed this operated through NFκB or MYD88 pathways, respectively. The enthesis pDC TNF induction by TLR7/9 is interesting since this is a critical cytokine in enthesitis (18). Although TNF is produced by many cells, pDCs was the sole entheseal source of TLR9 induced IFNα. The pDCs production of type-I interferons is a quintessential component of viral defense (19). Bacterial triggers are well defined in reactive arthritis and in experimental SpA and a role for viral infection is also reported in reactive arthritis and several epidemiological surveys have shown a link between viral triggers and PsA onset (20–22), but viral initiation is not commonly thought of as important in axial disease including in AS. In keeping with this, we report that SARS-CoV-2 infection resulted in PsA disease flares. Also, pDCs have previously been reported to be more abundant in PsA synovial fluid when compared to osteoarthritis or rheumatoid arthritis (23, 24). Blood pDCs are decreased in peripheral blood of PsA and showed chemotaxis towards PsA synovial fluid (24).

We also investigated several SpA and PsA disease relevant compounds for their ability to modulate entheseal IFNα induction. In agreement with previous blood derived pDC studies, both PDE4i and Tofacitinib attenuated ODN induced IFNα (25, 26); however, methotrexate did not. As TLR stimulation does not directly signal *via* JAK-STAT pathway, Tofacitinib attenuation of entheseal IFNα production could be due to reduced pDC TLR mRNA receptor expression, thus leading to decreased IFNα production as previously reported (25).

The limitations of this study include the relatively small sample numbers and the fact that functional analysis could only be done on the spinous process peri-entheseal bone. However, it still highlighted that upregulation of TNF and IFNα in stimulated entheseal pDCs that could be blocked with JAK and PDE4 inhibition. Studies of pDCs from sites of active enthesitis would also be desirable. Second, the time length of post-infection was different for each PsA patient so we cannot use a specific time period to indicate how long symptoms persisted after infection.

In conclusion, entheseal pDCs may represent a novel population that bridges SpA enthesis immunopathology and may be a population modified by JAK and PDE4 inhibition and also provides a novel mechanistic link between infection, including viral triggers and SpA associated arthropathies. Numerical and functional abnormalities have already been reported in other rheumatic diseases including systemic lupus erythematosus and rheumatoid arthritis but this is the first study to show resident pDCs at the normal enthesis, which offers new insights into how infectious triggers may precipitate SpA pathology (27, 28).

## Data Availability Statement

The raw data supporting the conclusions of this article will be made available by the authors, without undue reservation.

## Ethics Statement

The study protocol of the current investigation was approved by North West-Greater Manchester West Research Ethics Committee. Written informed consent to participate in this study was provided by the participants’ legal guardian/next of kin.

## Author Contributions

QZ, CB and DM had substantial contributions to study conception and design. QZ, JV, YE-S, CB, and DM had substantial contributions to analysis and interpretation of data. All the authors had substantial contributions to acquisition of data, drafting the article or revising it critically for important intellectual content and final approval of the version of the article to be published.

## Funding

DM is funded by the Leeds NIHR Biomedical Research Centre. HR, TR, and CB are funded by the Novartis UK-investigator-initiated non-clinical research funding support. QZ, AW, and KS were funded by the Celgene supported PARTNER fellowship program. CB was partially funded by Pfizer who also provided Tofacitinib as a research compound.

## Conflict of Interest

The authors declare that the research was conducted in the absence of any commercial or financial relationships that could be construed as a potential conflict of interest.
