# Supplementary material for: SARS-CoV-2 Infection Induces Psoriatic Arthritis Flares and Enthesis Resident Plasmacytoid Dendritic Cell Type-1 Interferon Inhibition by JAK Antagonism Offer Novel Spondyloarthritis Pathogenesis Insights
Source: Front Immunol. 2021 Apr 15;12:635018. doi: 10.3389/fimmu.2021.635018 (PMC8082065; doi:10.3389/fimmu.2021.635018)
Supplement: Supplementary file 1 [file DataSheet_1.docx]

**Supplementary Material**

**Description of Entheseal plasmacytoid dendritic cells and their antagonisim with JAK inhibition as a novel pathway in Spondyloarthritis related inflammation**

**Supplementary Figure 1**

**Morphology of pDC and production of IFN𝜶 from stimulated pDC.**


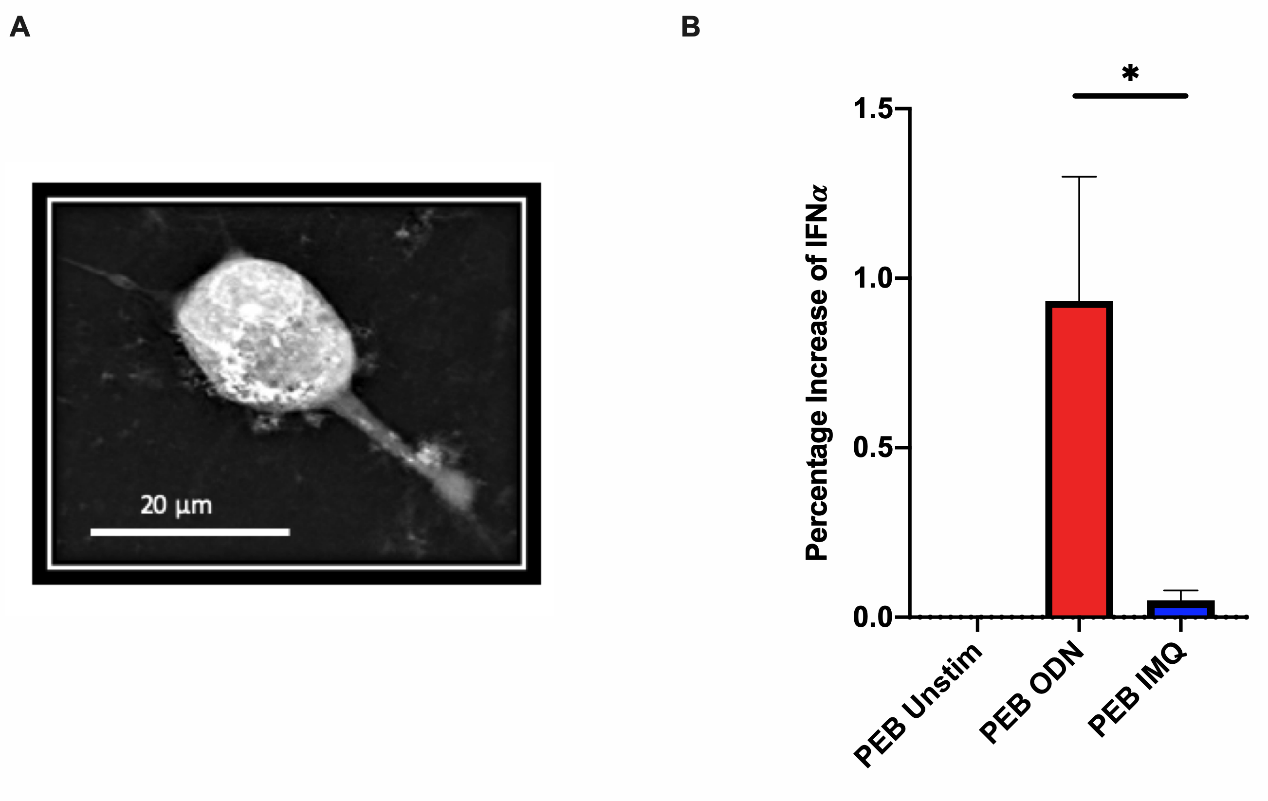


(A) The morphology of the entheseal pDC was confirmed using a holotomographic microscope (3D Explorer, NanoLive, Lausanne, Switzerland) with image taken at ×600 magnification. (B) PDC were stimulated with ODN or imiquimod and IFN𝜶 was measured by intracellular flow. Percentage increase over unstimulated cells was recorded. It was shown that percentage increase of IFN𝜶 after ODN stimulation was much higher than that following imiquimod stimulation (0.93±0.37% vs 0.05±0.29%, n=4, p=0.029). PEB: peri-entheseal bone, MFI: median fluorescence intensity, IMQ: imiquimod. Unstim: unstimulated. *: p<0.05.

**Supplementary Figure 2 Molecule activity predictor in dendritic cell maturation pathway obtained by IPA.**


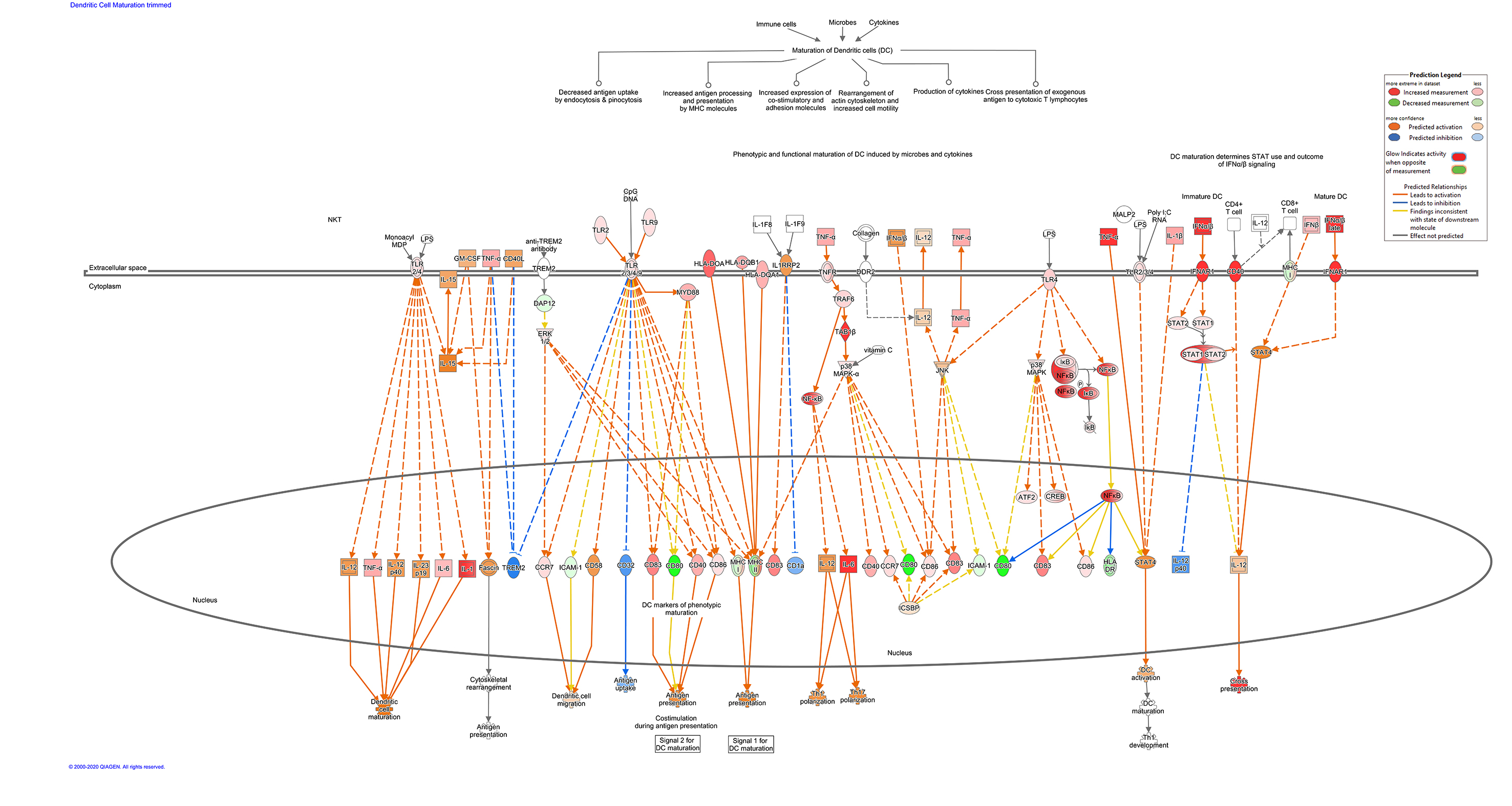


Ingenuity Pathway Analysis of entheseal dendritic cell maturation following ODN stimulation indicating net effect of TLR-9 mediated signalling following stimulation was the production of several proinflammatory cytokines, including TNF (upregulated, p=0.04, fold change=1.95), and adhesion molecules. TLR-mediated signalling is also predicted to be functionally interconnected with MYD88 and NF-κB with TNF, as a root regulator, further activating STAT4. Green represents downregulation e.g., CD40 and HLA-DR, while red & pink represents upregulation e.g., TAB1β & HLA-DOA, and intensity represents the relative magnitude of change in gene expression. Predicted activation is indicated by orange colour and blue for the predicted inhibition. Direct and indirect interactions are indicated by solid and dashed lines, respectively with these collectively leading to dendritic cell maturation and antigen presentation.

**Supplementary Figure 3. NF-κB signalling canonical pathway obtained by IPA.**

**
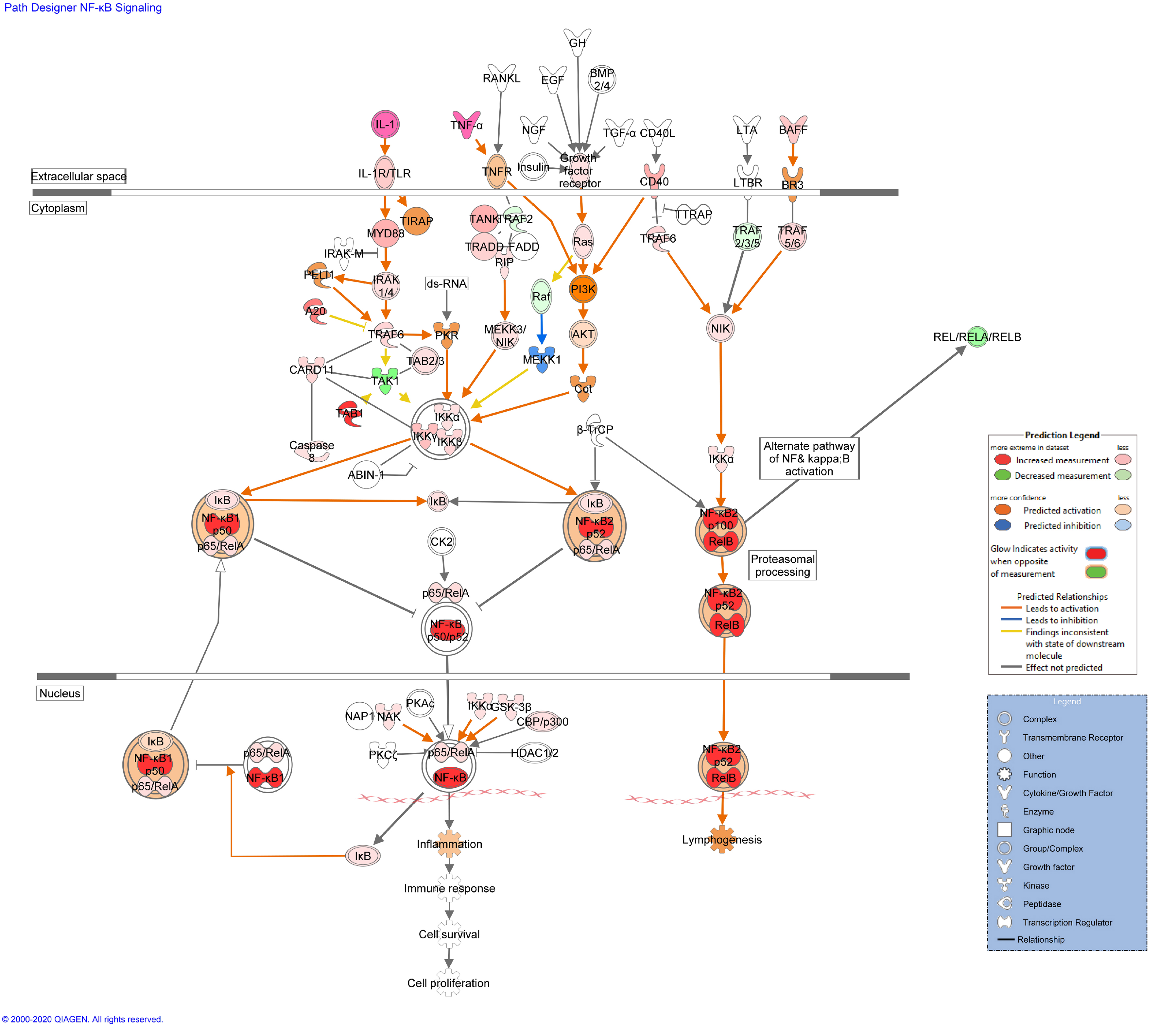
**

IPA predicts NF-κB canonical pathway activation after entheseal pDC stimulation with ODN. This network centers on the NF-κB transcription factor complex which mediate signals relevant to inflammation in response to TNF𝜶, IL-1 and other stimuli via PI3K, MYD88, or IKK𝜶 pathways, respectively. TLR7 (p=0.02, fold change=4.23) was significantly upregulated, thus a positive feedback loop is formed to enhance inflammation. Green represents downregulation while red & pink represent upregulation and intensity represents the relative magnitude of change in gene expression. Predicted activation indicated by orange colour and blue for the predicted inhibition. Direct and indirect interactions are indicated by solid and dashed lines, respectively.
